# Social Determinants of Health-Related Quality of Life among Residents in Zhejiang and Qinghai, China

**DOI:** 10.3390/ijerph16081314

**Published:** 2019-04-12

**Authors:** Yuxuan Gu, Hao Zhang, Shahmir H. Ali, Minzhuo Huang, Jingming Wei, Shuyan Gu, Xuemei Zhen, Xiaoqian Hu, Xueshan Sun, Hengjin Dong

**Affiliations:** 1 Center for Health Policy Studies, Department of Social Medicine, School of Public Health, Zhejiang University School of Medicine, 866 Yuhangtang Road, Hangzhou 310058, China; ygu33@jhu.edu (Y.G.); huangminzhuo@zju.edu.cn (M.H.); weijm@zju.edu.cn (J.W.); gushuyan@zju.edu.cn (S.G.); zhenxuemei@zju.edu.cn (X.Z.); huxiaoqian@zju.edu.cn (X.H.); sunxueshan@zju.edu.cn (X.S.); 2 Medical College, Hangzhou Normal University, Hangzhou 310058, China; zju_med@126.com; 3 The Johns Hopkins Bloomberg School of Public Health, Baltimore, MD 21218, USA; sali43@jhu.edu

**Keywords:** EQ-5D, health-related quality of life, population health, regional differences, China

## Abstract

Social determinants are closely related to health and play a significant role in shaping the quality of life of a population. This study aimed to explore the differences in HRQoL (health-related quality of life) scores of residents in the eastern province of Zhejiang and the western province of Qinghai and probe factors affecting the HRQoL among the two populations. A sample of 4210 residents from a cross-sectional survey was included in the analysis. The EQ-5D-3L instrument was used to measure the HRQoL of residents. A Chi-square test and a t-test were used to examine the differences between different variables and analysis of variance (ANOVA) with interaction effects was used to analyze factors associated with the HRQoL between the two provinces. Residents’ EQ-5D index score (EQ VAS score) was 0.963 (82.71) and 0.962 (81.51), respectively, in Zhejiang and Qinghai. Generally, residents in Qinghai displayed significantly worse HRQoL scores than those in Zhejiang. The differences between the two regions lay on mobility, pain/discomfort, and anxiety/depressions. In both regions, an increased education level and being employed were most strongly associated with a positive HRQoL; increased age and presence of chronic diseases were most strongly associated with a negative HRQoL. When formulating health policies, the significant health disparities between western and eastern provinces must be given greater consideration. The health of vulnerable groups should be particularly focused on to improve the observed health disparities.

## 1. Introduction

Disparities in population health exist not only between countries, but also within a country [1]. Several social determinants, such as economic stability, education, social context, and health care, are closely related to health. Several studies have reported regional differences as an important factor related to health status and showed that inequalities in health status exist between different regions and areas [2].

As the world continues to experience an epidemiologic transition from an infectious disease burden to a more chronic disease burden, health-related quality of life (HRQoL) is gaining increased traction in fields of population health measurements [3,4,5]. With the development of social economies and the improvement of living standards, peoples’ concepts of health are changing continuously. Indeed, the World Health Organization (WHO) in 1948 went to the extent of defining health as, “a state of complete physical, mental and social well-being and not merely the absence of disease or infirmity” [6]. As a result, the measurement of health status has been a field of constant evolution. Indeed, such variables are reflected by the WHO’s definition of quality of life as “an individual’s perception of the state of life in the context of the culture and value systems in which they live” [7]. With these changing norms of quality of life, concepts, such as HRQoL, have materialized. HRQoL was defined as including five domains: Death and duration of life, impairment, health perceptions, opportunity (capacity for health), and functional status [8]. It includes physical and mental health perceptions (e.g., energy level, mood) and their correlates—including health risks and conditions, functional status, social support, and socioeconomic status. It reveals aspects of physical, mental, social, and emotional health. HRQoL can more closely reflect the subjective aspects of quality of life than measures, such as life expectancy. This study focused on the EuroQol five-dimensional (EQ-5D) scale, a standardized HRQOL questionnaire developed by the EuroQol Group to provide a simple, convenient, and generic measure of health for clinical and economic appraisal.

The social determinants of health are the conditions in which people are born, grow, live, work, and age. These circumstances are shaped by the distribution of money, power, and resources at global, national, and local levels. The social determinants of health are mostly responsible for health inequities—the unfair and avoidable differences in health status seen within and between countries [9]. Different social determinants of health play a significant role in shaping the quality of life of a population. However, importantly, the impact of many of these social determinants of health on quality of life may be directly influenced by aspects of the certain region or province people are residing in. For example, regions with strong social or economic support systems (as a result of greater political or economic capital) may work to mitigate the effect of factors, such as age or socio-economic status, on quality of life (as compared to regions without this safety net in which these determinants may have a greater role in influencing quality of life). China, with the world’s largest population, has various regional differences in standards of living, culture, and customs. Given the previously established links between certain health and social determinants, such as regional economy, culture, social environments, customs, socioeconomic status, living conditions, and behavior [10,11,12,13,14,15], the differences in these various social characteristics between different provinces in China can likewise be expected to have an influence on population health and quality of life between the two provinces.

Two examples of Chinese provinces with stark differences in various social and economic sectors are the provinces of Zhejiang and Qinghai. Zhejiang is an easterly province located on the southeast coast of China while Qinghai is a westerly province located in the northwest. Both provinces display stark differences in population density, culture, economic status, and social infrastructure. Zhejiang and Qinghai have a total population of 55.9 million and 5.93 million, respectively. Per capita gross domestic product (GDP) in Zhejiang was 84,528 RMB while in Qinghai it was 43,380 RMB [16]. Since these are two different geographic provinces in China with differing structural features, residents are very likely to have different HRQoL scores and, importantly, different influencing social factors need to be identified in different regions for better interventions for a better quality of life. To the best of our knowledge, no study had compared the HRQoL of ordinary people between an eastern and western province in China specifically [17,18,19,20,21]. 

This study aimed to take Zhejiang and Qinghai as two cases to compare the HRQoL status of ordinary people between two regions, and to explore factors associated with HRQoL in these two respective regions. Based on the literature, we hypothesize that there is a significant difference between the two provinces and that certain social determinants will indeed show differing levels of influence between Zhejiang and Qinghai due to the systematic structural, social, and cultural differences between these provinces. By identifying the social determinants that are more associated with HRQoL in one province over the other, we are seeking to provide insights into more targeted interventions to improve the quality of life in different regions in China. 

## 2. Materials and Methods 

### 2.1. Study Sample

Our study sample was derived from a health service household survey of residents in Zhejiang and Qinghai. A multi-stage stratified cluster random sampling method was used to obtain 4901 participants during 2016 and 2017. 

**Figure 1:**
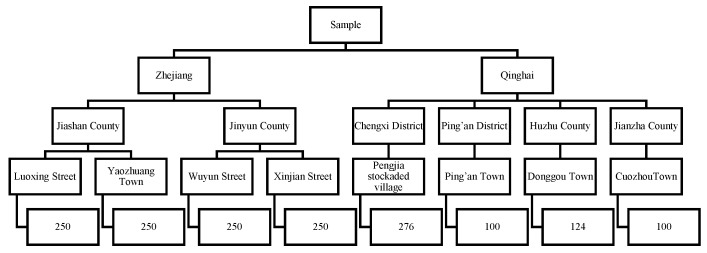
Sampling and sample size.

The sample was derived from the resident population in the region, including the residents living in the region for the past six months and migrant populations living in the region for more than half of each year. All ages were considered in the study sample. Multi-stage stratified cluster random sampling was used to select samples. First, Zhejiang and Qinghai were selected from half of the wealthiest and least wealthy provinces, respectively. Second, for both Zhejiang and Qinghai, counties were divided into two groups based on per capita wealth. In Zhejiang, one county was selected from each of the subgroups: Jiashan County (higher per capita wealth) and Jinyun County (lower per capita wealth). In Qinghai, due to the low per-county population density, two counties were selected from each of the subgroups: Chengxi district, Ping’an district (higher per capita wealth) and Huzhu county, Jianzha county (lower per capita wealth). Next, for each county, a town was randomly selected from an urban area and a street or town was randomly selected from a rural area. Finally, a sample of households was taken from each town/street. For this study, the characteristics of individual household residents from this household population were analyzed. A diagram of the described sampling method with the sample size is displayed in Figure 1.

### 2.2. Data Collection

Face-to-face interviews were conducted by trained investigators who used the health service household questionnaire in the survey. The questionnaire included individual socioeconomic status, demographic characteristics, residents’ health status (EQ-5D), and utilization of health services. Each questionnaire included a section of the standard EQ-5D-3L survey. The investigators were trained as a group on uniform standards and passed the coincidence examination. According to the requirements of the EQ-5D scale with regard to practical applications, residents were required to be 15 years of age or older and answer the questions by themselves [22]. The investigators were local personnel chosen by health-affiliated departments, such as the local health commission and the local center for disease control and prevention. After obtaining informed consent, all members of a household were interviewed in their homes individually. They were told to answer truthfully and that there was no right or wrong answer. Quality control was performed by the professors and students of Zhejiang University. Ethical approval was obtained from the institutional review board of Zhejiang University School of Medicine (2015022). Participant data remained anonymous throughout the research process.

This study only used the general household, individual variables, and the EQ-5D information. This information included (1) household’s data: Household income; (2) individual demographic and socioeconomic data: Age, sex, marital status, ethnic group, residence, basic insurance, the status of chronic diseases, education level, and occupational status; (3) individual self-reported health status based on the EQ-5D descriptive system and visual analogue scale (VAS). 

### 2.3. HRQoL Evaluation Instrument

The 3-level version of EQ-5D was introduced in 1990 by the EuroQol Group. It essentially consists of 2 parts: The EQ-5D descriptive system and the EQ visual analogue scale (EQ VAS). Value sets were generated using the time trade-off (TTO) valuation technique.

#### 2.3.1. EQ-5D-3L Descriptive System

The EQ-5D-3L descriptive system comprises the following five dimensions: Mobility, self-care, usual activities, pain/discomfort, and anxiety/depression. Each dimension has 3 levels: No problems, some problems, and extreme problems. The patient is asked to indicate his/her health state by ticking the box next to the most appropriate statement in each of the five dimensions. This decision results in a 1-digit number that expresses the level selected for that dimension. The digits for the five dimensions can be combined into a 5-digit number that describes the patient’s health state. The digits for the five dimensions can be combined into a 5-digit number that describes the patient’s health state. Based on the calculation using the Chinese EQ-5D scale utility value integral system, the EQ-5D index score ranges from −0.149 to 1.00.

#### 2.3.2. EQ VAS

The EQ VAS records the residents’ self-rated health on a vertical visual analogue scale where the endpoints are labelled ‘best imaginable health state’—100 and ‘worst imaginable health state’—0. The VAS can be used as a quantitative measure of health outcome that reflects the patient’s own judgement.

### 2.4. Analysis Methods

In the survey, the residents interviewed were in all age groups. Due to the requirement of participants being aged 15 years or older and providing responses to the EQ-5D section by themselves, a sample of 4231 was included. Data was further excluded if there were missing values for sex, age, ethnic group, residence, marital status, education level, employment, household income, health insurance, and chronic diseases, or the 5 dimensions of the EQ-5D or EQ VAS score. After the process of data cleaning, the effective sample size was 4210. Among them, 2530 were from Zhejiang and 1680 were from Qinghai. All data was corroborated using EpiData version 3.1 (The EpiData Association, Odense, Denmark), and then analyzed with R version 3.4.3 (R Foundation for Statistical Computing, Vienna, Austria). The significance level was *p* < 0.05.

#### 2.4.1. EQ-5D Calculation

The EQ-5D health utility value was calculated using the Chinese EQ-5D scale utility value integration system. The utility value integration system is shown in Table 1. C is a constant term, and MO2, SC2, UA2, PD2, and AD2 indicate that if mobility, self-care ability, daily activity, pain/discomfort, and anxiety/depression are at level 2, the value, 1, is assumed, and the other level is 0; MO3, SC3, UA3, PD3, and AD3 indicate that the above dimensions are 1 when at level 3 and 0 at others; N3 indicates that at least 1 of the 5 dimensions is 1 when at level 3. According to the results of Table 1, the utility values of all 243 health states can be calculated. The answers to the EQ-5D-3L questionnaire in this survey were converted into a utility index score through this Chinese general population-based EQ-5D-3L social value set. Details of the calculation were described previously [23]. The higher the EQ-5D index score and EQ VAS score, the better is the respondent’s HRQoL.

#### 2.4.2. Analysis of Influencing Factors of HRQoL

The differences between different variables, including demographic variables and the five dimensions of the EQ-5D health status, in Zhejiang and Qinghai were evaluated using the Chi-square test. For continuous variables, a t-test was conducted. Considering the significantly different sociodemographic characteristics in the two regions, an analysis of variance (ANOVA) with interaction effects and simple effects tests were used to analyze the influencing factors. Also, a procedure for alpha adjustment using Bonferroni’s procedure was performed. In measuring HRQoL, an individual’s subjective health feelings may be more reflective of an individual’s health level. Also, considering that there is no difference in the EQ-5D index score between Zhejiang and Qinghai, we focused on the influencing factors of the EQ VAS score. The results of the initial ANOVA model were used to determine statistically significant interaction terms of the province and sociodemographic variables, which were then the focus of a further pairwise comparison analysis.

We chose the variables based on the conducted literature review. Independent variables in the model include sex, age, ethnic group, residence, marital status, education level, occupation, income, basic health insurance, and conditions of chronic diseases. The variable coding can be seen in Table 2. As for income groups, residents were ranked from lowest to highest by their annual income and divided into five groups of equal size: The lowest income group had an income level below 5000 RMB; the second group from 5001 to 10,000 RMB; the third group from 10,001 to 20,000 RMB; the fourth group from 20,001 to 32,000 RMB; and the fifth and highest income group 32,001 from RMB and above.

## 3. Results

### 3.1. Demographic Characteristics of the Sample

The characteristics of the study sample are summarized in Table 2. In the sample, there were 2088 males (49.6%) and 2122 females (50.4%) in total. The mean age was 46.08 (±17.455). Of the residents, 88.6% were Han and the rest were other ethnic groups, including Tu, Tujia, Tibetan, Hui, Miao, etc. Among them, almost half lived in rural areas and half lived in urban areas. With respect to marital status, 3180 (75.5%) of them were married, 726 (17.2%) of them were never married, and 304 (7.2%) of them were divorced or widowed. Per capita household income was calculated and divided into five groups, with most people being categorized in the middle groupings. With regards to educational status, most people obtained a highest education level of junior middle school, followed by primary school, and individuals with college and above levels only accounting for 8.6% of the study population. Among them, worker/clerk and farmer accounted for more than half (57.2%) of the total population. In terms of basic health insurance, 97.9% residents enrolled in the basic health insurance. Of the residents, 15.3% had chronic diseases (Table 2). Overall, residents in Zhejiang and Qinghai have significant differences in age, ethnic group, residence, marital status, per capita household income, education, employment, and conditions of chronic diseases.

### 3.2. Health Profiles and EQ VAS Score

In terms of the EQ-5D index score and EQ VAS score, there was a significant difference in the EQ VAS score, but this difference was not found in the EQ-5D single summary index (Table 3). In other words, residents in Zhejiang and Qinghai indeed displayed differences in health status, but more through a patient’s own assessment of health status rather than the general population health.

Furthermore, although there was no difference in the overall EQ-5D index score, there were still differences in the five dimensions of health. Table 3 also shows the horizontal components of each dimension and compares the differences between Zhejiang and Qinghai. It can be seen that residents in Qinghai have a higher proportion of moderate/severe problems than residents in Zhejiang in terms of mobility and anxiety/depression. In pain/discomfort, people in Qinghai had more severe problems, and people in Zhejiang had more moderate problems. According to the results of the Chi-square test, the differences in the composition of the above three components between residents in Zhejiang and Qinghai were statistically significant.

With regards to the difference of the EQ VAS score between the two regions, the EQ VAS score by sociodemographic variables were reported (Table 4). The mean EQ-VAS scores were 82.71 (13.05) for residents in Zhejiang and 81.51 (13.92) for residents in Qinghai overall, indicating that the HRQOL of residents in Zhejiang was better than that of Qinghai (*p* < 0.01). Moreover, in most subgroup analyses (only the variables with statistical significance are displayed), the EQ-VAS scores of residents in Zhejiang were higher than those of residents in Qinghai with statistically significant differences. Only residents in Zhejiang who earned more than 20,001 Yuan annually (*p* < 0.05) had lower EQ-VAS scores compared with the residents in Qinghai in that category (Table 4).

### 3.3. Factors Influencing HRQoL

The results of the analysis of variance (ANOVA) with interaction effects model created are summarized in Table 5. Looking at the socio-demographic variables across the total study population, an increase in education level was strongly associated with an improved EQ VAS score. Being employed was significantly associated with a higher EQ VAS score compared with those unemployed. Residents living in rural areas had a better health status than those in urban areas. Residents who were covered by basic insurance had a worse health status than those who were not. Aging and having chronic diseases were significantly negatively associated with health status in the two regions. As age increased, people’s health status grew worse. 

Of the nine interaction terms (between the binary province variable and the socio-demographic variables) included in the model displayed in Table 5, only three interaction variables were statistically significant and selected for further analysis: Province × ethnic group, province × marital status, and province × income. With these significant interaction terms, pairwise comparisons were conducted both between different levels within each socio-demographic variable in each respective province (Table 6) and the value of each level within each socio-demographic variable between the two provinces (Table 7). 

In Zhejiang, we found that residents who were not of Han ethnicity or divorced/widowed had significantly lower EQ VAS scores. Among the residents in Qinghai, having a higher income was significantly associated with higher EQ-VAS scores (Table 6). When compared by province, people who were divorced/widowed, had a household income between 20,001 and 32,000 yuan per year, or a household income higher than 32,000 yuan per year had significantly lower EQ VAS scores in Zhejiang than in Qinghai (Table 7).

## 4. Discussion

With continuous improvements in life expectancy, the examination of other indicators, such as HRQoL, of residents in China is being explored by health-related researchers. Health disparities widely exist among different regions in China [24].

In this study, we examined and compared the HRQoL measured by the EQ-5D 3L questionnaire and explored factors influencing discrepancies in quality of life in Zhejiang and Qinghai. The results provide useful policy suggestions to the regional population health in China. Although it was found that the trend in the EQ VAS scores showed statistically significant relationships, no significance in the EQ-5D index scores was found. As expected, the health status of residents in Zhejiang was better than in Qinghai. 

From the results of the study, among the five dimensions in residents in Zhejiang and Qinghai, the most prevalent problems are pain/discomfort and anxiety/depression, and these results are aligned with the results of the Fifth National Health Service Survey in China and other research results in China, but are significantly lower than that in the United States, Britain, or Australia, which may be related to the different levels of economic development in the different countries. Moreover, after comparing the two regions, we found that the proportion of residents in Qinghai who have self-reported problems in the dimensions of mobility and anxiety/depression are significantly higher than that of residents in Zhejiang. In terms of pain/discomfort, residents of both populations display a need for future targeted interventions; in Zhejiang, there are more residents with moderate problems, but fewer residents with severe problems in Zhejiang, which may indicate that residents with moderate problems may become more severe later in Zhejiang, while the Qinghai population displayed a higher proportion of severe problems. Anxiety/depression reflects problems in the areas of psychology, and residents in Qinghai displayed more severe psychological problems than residents in Zhejiang. 

The mean EQ VAS score of residents in Zhejiang was significantly higher than residents in Qinghai. The mean of the EQ-5D score of residents in Qinghai was lower than residents in Zhejiang (albeit without statistical significance). These results indicate that the HRQoL of residents in Zhejiang is better than that of residents in Qinghai. These findings can be explained by the fact that Qinghai and Zhejiang have a significant difference in socioeconomic development, which has a large correlation with respondents’ health status. Indeed, these results shed crucial light onto why residents in Zhejiang display metrics of significantly higher quality of life than those in Qinghai.

According to the results of the analysis of variance (ANOVA) with interaction effects and pairwise comparisons of simple effects, there is a significant positive correlation between socioeconomic status and HRQoL in these two areas. The higher the income level, the higher the level of education, and the more employed, the better the HRQoL. Thus, this study suggests the need for further development of public policies that are conducive to the poor, narrowing the poverty gap, and at the same time raising the overall level of national education. Age is also one of the important factors affecting HRQoL. The health of people over the age of 15 was shown to gradually decline with increases in age, which is consistent with the expected assumption. Basic health insurance policies for residents should continue to be given more attention because of the possible impact on the health status of residents. Notably, the condition of chronic diseases of residents always has an impact on residents’ health status. This indicates that the status of physical health is still a notable problem and residents need to pay attention to individual health behaviors, such as drinking, smoking, physical activity, and diet. Specifically, in Zhejiang, we found that residents who were not of the Han ethnic group, and were divorced or widowed had significantly lower EQ VAS scores. The health status of the majority Han ethnic group was better than minority groups, indicating that minorities are in a relatively disadvantaged state of health in Zhejiang. Among residents in Qinghai, having a higher income was significantly associated with higher EQ-VAS scores, which indicates policies should still focus on improving the economic level and income in Qinghai. When compared by province, residents in Zhejiang who were divorced or widowed had significantly lower EQ VAS scores than residents in Qinghai. Residents with a higher income had lower EQ VAS scores in Zhejiang than Qinghai, which indicates that measures taken to improve household income levels may help to improve the quality of life among residents in Qinghai to a greater extent than residents in Zhejiang. Compared to other similar studies, several factors were also found to be significant social determinants of quality of life in other jurisdictions and sub populations. A study conducted in Iran showed that determinants of HRQoL included monthly household income, post-secondary education, age, marriage status, and having chronic diseases [25]. A study conducted in China using a short form 36 health survey questionnaire (SF-36) questionnaire to evaluate HRQoL in the general population showed that risk factors included aging, past history of chronic disease, occupations, such as farming and being a student, and gender, while protective factors included marriage and higher education [26]. However, in this study, it must be noted that the EQ VAS score is a relatively subjective evaluation indicator for health status and the difference in health in practical terms is still worthy of further investigation.

These results have some important implications for population health in China. First, not only physical health, but also mental health should be noted, especially in the western area. Also, different places have different health profiles and a different proportion of risk factors, which should be treated in corresponding ways.

There are also some key limitations of this study. First, as a cross-sectional study, it is difficult to establish correlational or causational relationships due to the weak strength of the data in comparison with longitudinal formats. Second, when compared by different regions, we should consider that the EQ-5D value may have a ceiling effect and the definition of the top and bottom anchors on the EQ VAS is vague [27].

In addition, the results of HRQoL in our study were similar to former studies conducted in China, supporting the consistency of our data [2,17,18,20,21]. From a global context, much of the HRQoL results were observed to be higher or lower depending on the socio-demographic variables compared to studies conducted in settings, such as the U.S., Europe, and Vietnam [28,29,30]. However, as noted, making inter-country comparisons is an area that needs to be further investigated given the high levels of subjectivity associated with the HRQoL measure. Since the EQ-5D instrument is a self-perceived health status measure, it may vary among people not only by their actual health status, but also by different cultural backgrounds, by the expectations of their health status, and by the education level of the population. Therefore, people with different nationalities and cultural backgrounds with the same health status may still give their health status a different rating [31].

## 5. Conclusions

The HRQoL of residents in Zhejiang was better than that of residents in Qinghai. Residents in Qinghai had a higher proportion of health problems than residents in Zhejiang in terms of mobility and anxiety/depression. The main common factors affecting HRQoL include age, residence, occupation, education, and the condition of chronic diseases. To improve the HRQoL of residents, raising the education level and improving the quality and accessibility of health services are two important measures. Also, between different regions, slightly different interventions should be considered, for example, the improvement of income level is an independent positive factor influencing residents in Qinghai. In addition, apart from improving the accessibility of medical services, it is also important to strengthen interventions in psychological issues, especially in a western region, like Qinghai.

## Figures and Tables

**Table 1:** Chinese EuroQol five-dimensional (EQ-5D) scale utility value integral system.

C	MO2	MO3	SC2	SC3	UA2	UA3	PD2	PD3	AD2	AD3	N3
0.039	0.099	0.246	0.105	0.208	0.074	0.193	0.092	0.236	0.086	0.205	0.022

**Table 2:** Characteristics of the sample.

Characteristic	Total (*n* = 4210)	Zhejiang (*n* = 2530)	Qinghai (*n* = 1680)	*p*
Sex (%)				0.743
Male	2088 (49.6)	1260 (49.8)	828 (49.3)	
Female	2122 (50.4)	1270 (50.2)	852 (50.7)	
Age (Mean/SD)	46.08 (17.46)	47.98 (17.44)	43.21 (17.08)	
Age group (%)				<0.001
15–	552 (13.1)	250 (9.9)	302 (18.0)	
25–	635 (15.1)	370 (14.6)	265 (15.8)	
35–	771 (18.3)	461 (18.2)	310 (18.5)	
45–	967 (23.0)	582 (23.0)	385 (22.9)	
55–	618 (14.7)	401 (15.8)	217 (12.9)	
65 or more	667 (15.8)	466 (18.4)	201 (12.0)	
Ethnic group (%)				<0.001
Han	3732 (88.6)	2493 (98.5)	1239 (73.8)	
Others	478 (11.4)	37 (1.5)	441 (26.3)	
Residence (%)				<0.001
Rural	2382 (56.6)	1550 (61.3)	832 (49.5)	
Urban	1828 (43.4)	980 (38.7)	848 (50.5)	
Marital status (%)				<0.001
Never married	726 (17.2)	342 (13.5)	384 (22.9)	
Married	3180 (75.5)	2012 (79.5)	1168 (69.5)	
Divorced or widowed	304 (7.2)	176 (7.0)	128 (7.6)	
Average household income (%)				<0.001
≤5000	955 (22.7)	223 (8.8)	732 (43.6)	
5001–	814 (19.3)	348 (13.8)	466 (27.7)	
10,001–	1007 (23.9)	715 (28.3)	292 (17.4)	
20,001–	594 (14.1)	496 (19.6)	98 (5.8)	
>32,000	840 (20.0)	748 (29.6)	92 (5.5)	
Education (%)				<0.001
Below primary school	466 (11.1)	156 (6.2)	310 (18.5)	
Primary school	943 (22.4)	649 (25.7)	294 (17.5)	
Junior middle school	1322 (31.4)	810 (32.0)	512 (30.5)	
Senior middle school	728 (17.3)	433 (17.1)	295 (17.6)	
Junior College	391 (9.3)	239 (9.4)	152 (9.0)	
College and above	360 (8.6)	243 (9.6)	117 (7.0)	
Employment status (%)				<0.001
Administrator and professional	664 (15.8)	471 (18.6)	193 (11.5)	
Worker/Clerk	1266 (30.1)	892 (35.3)	374 (22.3)	
Farmer	1143 (27.1)	503 (19.9)	640 (38.1)	
Student	301 (7.1)	123 (4.9)	178 (10.6)	
Unemployed or semi-employed	601 (14.3)	318 (12.6)	283 (16.8)	
Other	235 (5.6)	223 (8.8)	12 (0.7)	
Basic health insurance (%)				0.394
Yes	4120 (97.9)	2472 (97.7)	1648 (98.1)	
No	90 (2.1)	58 (2.3)	32 (1.9)	
Chronic diseases (%)				<0.001
Yes	645 (15.3)	341 (13.5)	304 (18.1)	
No	3565 (84.7)	2189 (86.5)	1376 (81.9)	

**Table 3:** Five dimensions of EQ-5D and visual analogue scale (VAS) score.

EQ-5D & VAS	Zhejiang (*n* = 2530)	Qinghai (*n* = 1680)	*p*
Mobility (%)			<0.05
No problem	2424 (95.8)	1596 (95.0)	
Moderate problems	100 (4.0)	70 (4.2)	
Severe problems	6 (0.2)	14 (0.8)	
Self-care (%)			0.179
No problem	2472 (97.7)	1633 (97.2)	
Moderate problems	49 (1.9)	34 (2.0)	
Severe problems	9 (0.4)	13 (0.8)	
Usual activities (%)			0.065
No problem	2428 (96.0)	1605 (95.5)	
Moderate problems	84 (3.3)	51 (3.0)	
Severe problems	18 (0.7)	24 (1.4)	
Pain/discomfort (%)			<0.001
No problem	2163 (85.5)	1529 (91.0)	
Moderate problems	352 (13.9)	127 (7.6)	
Severe problems	15 (0.6)	24 (1.4)	
Anxiety/depressions (%)			<0.05
No problem	2404 (95.0)	1563 (93.0)	
Moderate problems	118 (4.7)	107 (6.4)	
Severe problems	8 (0.3)	10 (0.6)	
EQ-5D index score (mean/SD)	0.963 (0.104)	0.962 (0.125)	0.869
EQ VAS score (mean/SD)	82.71 (13.05)	81.51 (13.92)	<0.01

**Table 4:** Health-related quality of life by sociodemographic variables.

Variables	Zhejiang	Qinghai	*p*
Sex (Mean/SD)			
Male	83.21 (12.985)	81.91 (13.223)	<0.05
Female	82.23 (13.106)	81.12 (14.557)	0.067
Age group (Mean/SD)			
15–	92.18 (9.607)	88.89 (8.671)	<0.001
25–	89.51 (8.284)	87.08 (12.140)	<0.01
35–	86.39 (9.709)	83.18 (11.630)	<0.001
45–	82.55 (11.303)	80.56 (13.303)	<0.05
55–	78.87 (11.779)	75.30 (14.086)	<0.01
65 or more	72.10 (15.052)	69.03 (15.089)	<0.05
Residence (Mean/SD)			
Rural	82.81 (13.488)	80.88 (14.056)	<0.01
Urban	82.57 (12.339)	82.13 (13.761)	0.474
Marital status (Mean/SD)			
Never married	90.68 (11.218)	86.55 (12.272)	<0.001
Married	82.25 (12.229)	80.69 (13.612)	<0.01
Divorced or widowed	72.51 (16.350)	73.87 (16.195)	0.474
Average household income (Mean/SD)			
≤5000	79.10 (15.689)	78.50 (15.406)	0.608
5001–	83.21 (15.277)	83.65 (11.859)	0.647
10,001–	82.79 (13.414)	83.17 (13.810)	0.687
20,001–	82.62 (12.146)	85.87 (7.767)	<0.05
>32,000	83.55 (10.991)	84.73 (11.752)	0.336
Employment status (Mean/SD)			
Administrator and professional	84.62 (10.484)	83.86 (12.489)	0.425
Worker/Clerk	84.72 (11.790)	84.88 (11.922)	0.824
Farmer	78.39 (13.836)	78.27 (13.942)	0.885
Student	92.24 (10.580)	89.38 (8.508)	<0.05
Unemployed or semi-employed	76.31 (15.369)	78.16 (16.302)	0.151
Other	84.30 (11.868)	73.33 (17.233)	<0.01
Basic health insurance (Mean/SD)			
Yes	82.62 (13.055)	81.37 (13.967)	<0.01
No	86.72 (12.409)	88.44 (8.747)	0.491

**Table 5:** ANOVA on the influencing factors and interacting factors. Dependent variable: VAS Score.

Source	Type III Sum of Squares	df	Mean Square	F	*p* Value
Corrected Model	231,605.525	53	4369.916	34.530	<0.001
Intercept	1,008,209.795	1	1,008,209.795	7966.578	<0.001
Province × Sex	3.698	1	3.698	0.029	0.864
Province × Age group	512.160	5	102.432	0.809	0.543
Province × Ethnic group	613.841	1	613.841	4.850	<0.05
Province × Residence	155.788	1	155.788	1.231	0.267
Province × Marital status	1808.859	2	904.430	7.147	<0.01
Province × Income	2098.080	4	524.520	4.145	<0.01
Province × Education	1318.789	5	263.758	2.084	0.064
Province × Occupation	1271.414	5	254.283	2.009	0.074
Province × Basic insurance	260.173	1	260.173	2.056	0.152
Province × Chronic diseases	145.116	1	145.116	1.147	0.284
Province	267.685	1	267.685	2.115	0.146
Sex	9.181	1	9.181	0.073	0.788
Age group	30,361.423	5	6072.285	47.981	<0.001
Ethnic group	319.236	1	319.236	2.523	0.112
Residence	556.232	1	556.232	4.395	<0.05
Marital status	847.111	2	423.556	3.347	<0.05
Income	1046.408	4	261.602	2.067	0.082
Education	5660.313	5	1132.063	8.945	<0.001
Occupation	2361.352	5	472.270	3.732	<0.01
Basic insurance	647.497	1	647.497	5.116	<0.05
Chronic diseases	31,920.800	1	31,920.800	252.229	<0.001
Error	525,962.352	4156	126.555		
Total	29,226,556.000	4210			
Corrected Total	757,567.876	4209			

R Squared = 0.306 (Adjusted R Squared = 0.297); A Bonferroni’s procedure was performed.

**Table 6:** Pairwise comparisons of interaction factors influencing the EQ VAS score (classified by province).

Province	Pairs	Mean Difference	Std. Error	95% CI	*p* Value
Zhejiang					
Ethnic group	Other-Han	−3.790	1.882	(−7.480, −0.100)	<0.05
Marital Status	Not married-Married	1.215	0.998	(−1.175, 3.605)	0.670
	Not married-Divorced or widowed	4.980	1.347	(1.754, 8.205)	<0.01
	Married-Divorced or widowed	3.764	0.941	(1.511, 6.018)	<0.001
Income	≤5000-5,001–	0.175	0.986	(−2.593, 2.943)	1.000
	≤5000-10,001–	0.918	0.906	(−1.627, 3.463)	1.000
	≤5000-20,001–	0.858	0.962	(−1.845, 3.561)	1.000
	≤5000->32,000	1.563	0.985	(−1.202, 4.329)	1.000
	5001–-10,001–	0.743	0.748	(−1.357, 2.842)	1.000
	5001–-20,001–	0.683	0.811	(−1.596, 2.961)	1.000
	5001–->32,000	1.388	0.818	(−0.910, 3.686)	0.900
	10,001-20,001–	−0.060	0.666	(−1.930, 1.811)	1.000
	10,001->32,000	0.645	0.654	(−1.191, 2.482)	1.000
	20,001->32,000	0.705	0.692	(−1.237, 2.648)	1.000
Qinghai					
Ethnic group	Other-Han	0.614	0.675	(−0.710, 1.938)	0.363
Marital Status	Not married-Married	−2.418	1.019	(−4.858, 0.022)	0.053
	Not married-Divorced or widowed	−2.553	1.469	(−6.071, 0.964)	0.246
	Married-Divorced or widowed	−0.136	1.118	(−2.813, 2.542)	1.000
Income	≤5000-5001–	−3.017	0.750	(−5.215, −1.000)	<0.001
	≤5000-10,001–	−2.443	0.893	(−4.951, 0.065)	0.063
	≤5000-20,001–	−4.713	1.354	(8.514, −0.911)	<0.01
	≤5000->32,000	−4.373	1.394	(−8.288, −0.458)	<0.05
	5001–-10,001–	0.664	0.861	(−1.754, 3.083)	1.000
	5001–-20,001–	−1.606	1.322	(−5.319, 2.107)	1.000
	5001–->32,000	−1.266	1.364	(−5.098, 2.566)	1.000
	10,001-20,001–	−2.270	1.346	(−6.051, 1.511)	0.919
	10,001->32,000	−1.930	1.384	(−5.816, 1.956)	1.000
	20,001->32,000	0.340	1.645	(−4.280, 4.960)	1.000

**Table 7:** Pairwise comparisons of interaction factors influencing the EQ VAS score (compared by province).

Independent Variables	Mean Difference (Zhejiang-Qinghai)	Std. Error	95% CI	*p* Value
Ethnic group				
Other	−4.787	2.452	(−9.595, 0.021)	0.051
Han	−0.383	1.517	(−3.357, 2.592)	0.801
Marital status				
Not married	1.137	2.037	(−2.856, 5.130)	0.577
Married	−2.496	1.803	(−6.031, 1.039)	0.166
Divorced or widowed	−6.396	2.188	(−10.686, −2.105)	<0.01
Income				
≤5000	1.046	1.938	(−2.755, 4.846)	0.590
5001–	−2.237	1.927	(−6.015, 1.541)	0.246
10,001–	−2.316	1.931	(−6.101, 1.470)	0.231
20,001–	−4.525	2.136	(−8.714, −0.337)	<0.05
>32,000	−4.891	2.144	(−9.094, −0.687)	<0.05
